# Intergenerational Learning: An Opportunity to Transform Nursing Students’ Perspectives of Older Adults

**DOI:** 10.1093/geroni/igab046.1264

**Published:** 2021-12-17

**Authors:** Marleen Thornton, Kathryn Burns, Lara Street

**Affiliations:** 1 Notre Dame of Maryland University, Baltimore, Maryland, United States; 2 Notre Dame of Maryland, Baltimore, Maryland, United States

## Abstract

The purpose of this pilot project was to explore the experience of an intergenerational learning environment focused on healthy aging for nursing students and older adults. Intergenerational learning experiences provide opportunities for individuals from different age groups to communicate and participate in learning activities together. The growing population of older adults calls for increased geriatric nursing expertise. Nursing students’ attitudes toward older adults are often negative though, and result in decreased interest in geriatric nursing. The opportunity to transform nursing students’ perspectives on older adults has the potential to improve nursing care for older adults, and the number of nurses focused on geriatric nursing care. This qualitative inquiry used a convenience sample of 10 participants from a cross-listed university course on healthy aging for baccalaureate nursing students and older adult members of a lifelong learning institute. Semi- structured focus group interviews were conducted. Narrative transcripts were analyzed using an inductive approach. Analysis illustrated improved nursing students’ perspectives of older adults and aging. A similar theme was noted for older adults’ perspectives of younger adults. The importance of social interaction within an intergenerational learning environment and the need for opportunities to challenge ageist perspectives was illustrated. Increased exposure to healthy older adults, personally and professionally, may increase nursing students’ interest in geriatric nursing and improve nursing care for older adults. Future research should examine more specifically how intergenerational learning experiences can decrease ageism, improve nursing students’ and nurses’ perspectives on older adults, and improve nursing practice for older adults.

